# Response to “in regard to “Tran A, Zhang J, Woods K, Yu V, Nguyen D, Gustafson G, Rosen L, Sheng K. Treatment planning comparison of IMPT, VMAT and 4π radiotherapy for prostate cases””

**DOI:** 10.1186/s13014-018-1010-5

**Published:** 2018-04-13

**Authors:** Ke Sheng

**Affiliations:** Department of Radiation Oncology, University of California, Los Angeles, 200 Medical Plaza, Suite B265, Los Angeles, CA 90095 USA

**Keywords:** 4π, Non-coplanar radiotherapy, Treatment planning, Optimization

## Abstract

In regard to our recently published paper entitled “Treatment planning comparison of IMPT, VMAT and 4π radiotherapy for prostate cases”, a question was raised whether “4π” was used appropriately to describe the non-coplanar planning and delivery space. In this letter, the term use is explained from both theoretical and practical perspectives. It is concluded that the self-explanatory term provides a flexible description of non-coplanar radiotherapy with beam orientation optimization. Confusions with this term can be avoided by understanding the evolving and machine/patient specific nature of 4π planning,

Letter to the Editor:

Recently, Dr. Sarkar questioned our use of “4π” to describe non-coplanar external beam radiotherapy. This discussion brought the much needed attention to this topic. However, I disagree with his conclusion that 4π should not be used to describe non-coplanar radiotherapy for the following reasons.

1. Dr. Sarkar’s assessment of the solid angles is mathematically incorrect. Without considering collision, Dr. Sarkar estimated the theoretical accessible solid angles based on the observation for a typical C-arm linac that the gantry has 360° access while the couch only has 180° access. The observation is correct but Dr. Sarkar neglected the symmetry of the C-arm gantry geometry and the additional freedom of combining the gantry and couch rotation. In fact, the couch only needs 180° rotation to provide the full 4π access. Figure 1 shows such a complete surface for a theoretical 4π treatment. One can visualize how the entire 4π solid angle is covered with the combination of 180° couch and 360° gantry rotation.
2. Even with the collision consideration, the angles that can be safely accessed for head or foot treatment is significantly larger than a hemisphere. One can consider a typical brain treatment where not only the superior angles are accessible, some of the inferior oblique angles are also available by rotating the gantry towards the patient torso while the couch is rotated. It is true that the solid angle for body treatment is more limited such as the left lung treatment shown in Fig 2a. When the source-to-isocenter distance is limited to 100 cm, the feasible angles shown in Fig 2c is limited. However, the accessible non-coplanar beam angles can be expanded by allowing source-to-isocenter distances greater than 100 cm. An example is shown in Fig. 2b using the validated geometrical modeling method described previously [1]. With the extended distances, approximately 70% of the 4π surfaces are covered for a left lung treatment. Note that a portion of the missing non-coplanar surface is caused by the couch pedestal occlusion.
3. Dr. Sarkar calculated the spherical surface covered by a 40 cm × 40 cm field in a 360° arc. He then argued that the actual solid angle is much smaller due to the smaller actual field sizes. This is a misunderstanding of 4π treatment planning, which indicates the freedom of *selecting* beams from the 4π solid angles instead of *using all* beams in a single plan.
4. There is a benefit of keeping 4π as an open software and hardware platform to encourage and incorporate future development. The mechanical restrictions to approach 4π solid angles are neither insurmountable nor static. Extended source-to-isocenter distances are readily achievable with existing C-arm platforms, although additional quality assurance and commissioning may be needed to safely use this mode. Robotic couches that are being introduced in the radiation oncology clinic [2] will certainly further expand the non-coplanar angle access if not enabling the full 4π solid angles. CyberKnife has limited posterior beam access but nothing fundamental prevents the next generation robotic linacs to gain access these angles.
5. Finally, there is a beauty in the nomenclature. 4π is not a typical undecipherable acronym that one has to look up. It is simple yet self-explanatory. Different from the conventional term of “non-coplanar radiotherapy”, it emphasizes the ability of automated beam orientation optimization in the non-coplanar solution space. It reflects continuously evolving effort in planning algorithms and delivery platforms advance radiotherapy dosimetry.

**Fig. 1:**
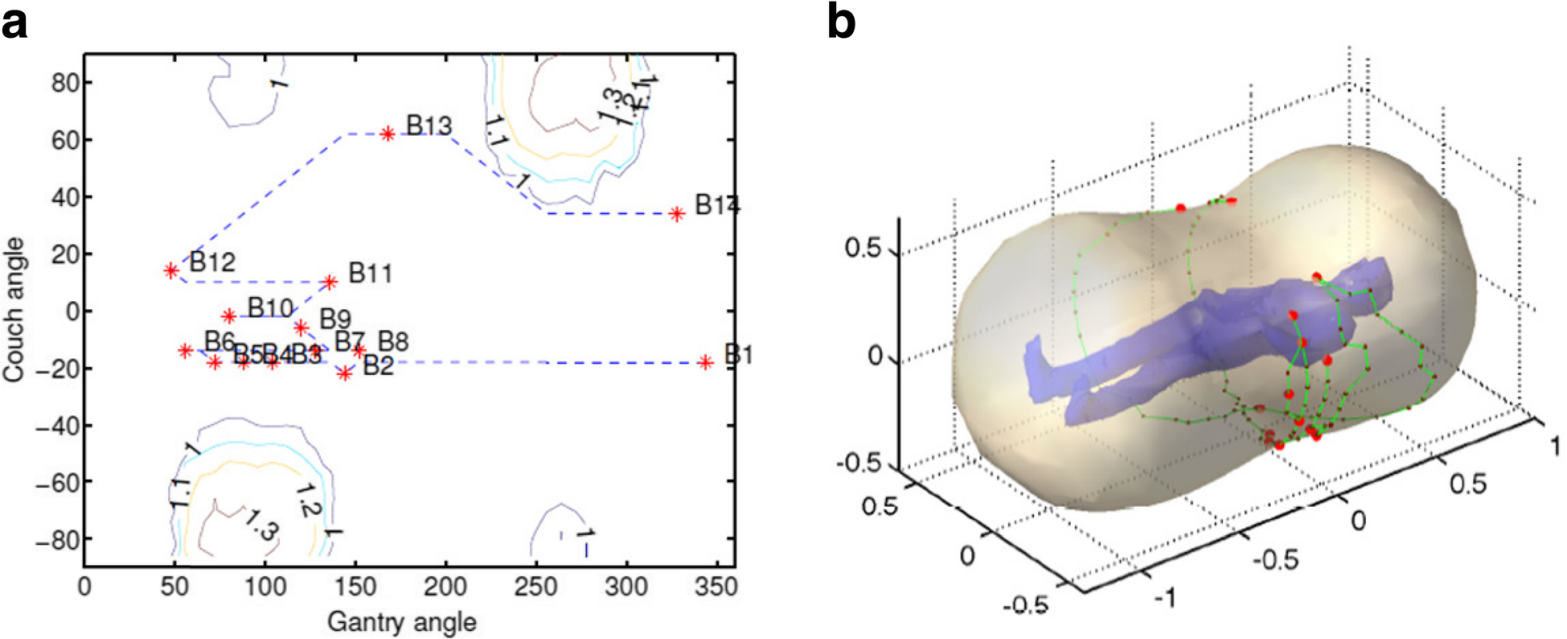
**a** Couch/gantry combination to achieve the entire 4π solid angle. The selected beams are denoted B1-B14. The isolines denote the minimal distances in meter between the source and the target to achieve those angles. The collision model only considered the patient surface and gantry without the couch in this case. **b** The corresponding patient centric view

**Fig. 2:**
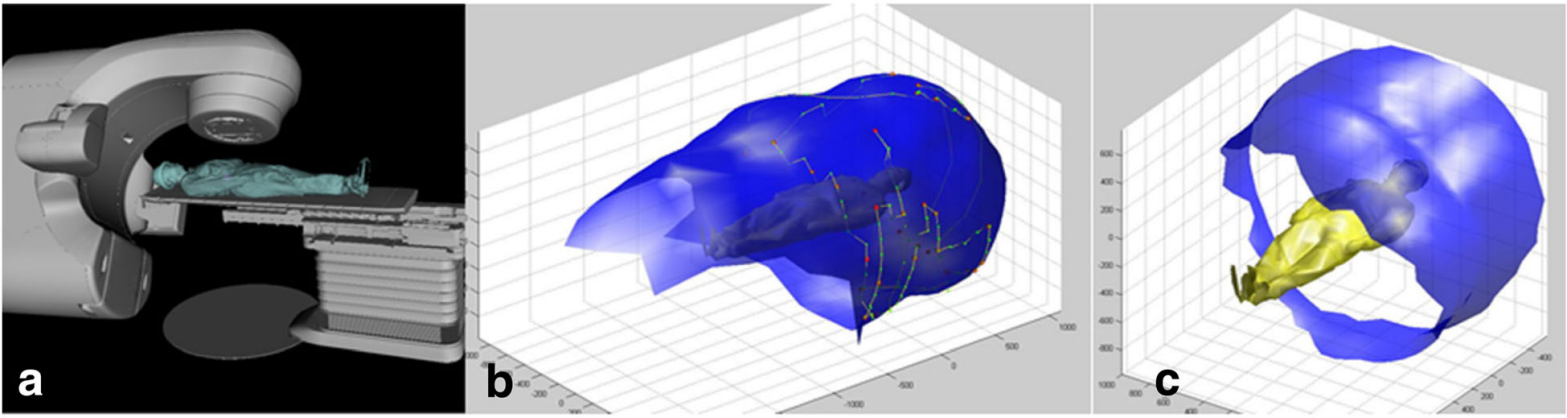
Accessible non-coplanar beam geometry for a left lung target based on the actual machine model and a 3D laser scanned human surface shown in (**a**). **b** The non-coplanar surface that can be accessed by the source when up to 120 cm source-to-isocenter distances are used by enabling additional couch shifts. **c** The non-coplanar surface that can be accessed by the X-ray source for treatment limited to 100 cm isocentric set up. The surface only accounts for a point source and that the effective coverage will be larger due to the typical 40 cm field-of-view

## References

[1] Yu VY, Tran A, Nguyen D (2015). The development and verification of a highly accurate collision prediction model for automated noncoplanar plan delivery. Med Phys.

[2] Baumeyer J, Vieyres P, Miossec S (2015). Robotic co-manipulation with 6 DoF admittance control: application to patient positioning in proton-therapy. 2015 Ieee international workshop on advanced robotics and its social impacts (Arso).

